# Thoracodorsal artery perforator flap as an autologous alternative to acellular dermal matrix

**DOI:** 10.1186/s12957-017-1254-9

**Published:** 2017-10-16

**Authors:** Tarek Hashem, Ahmed Farahat

**Affiliations:** 1 Department of Breast Surgery, National Cancer Institute, Cairo University, Cairo, Egypt; 2 Department of Surgical Oncology, National Cancer Institute, Nr 1.Fom el Khalig, Kasr el Aini str, Cairo, Egypt

**Keywords:** Breast reconstruction, Perforator flaps, Nipple-sparing mastectomy, Thoracodorsal artery, Subpectoral implants

## Abstract

**Background:**

Thoracodorsal artery perforator (TDAP) flap is one of the relatively new techniques in breast reconstruction. This pedicled flap retains the benefits of perforator flaps as regards minimal donor site morbidity without the need for microvascular anastomosis. Its role in partial breast reconstruction has been well documented. However, there are few reports about the role of this flap in total breast reconstruction.

**Methods:**

This study included 47 cases who presented to the breast unit of the National Cancer Institute of Cairo University from 2013 to 2015. All patients underwent nipple-sparing mastectomy with immediate implant-based reconstruction. The TDAP flap was used to complete the subpectoral pocket for the implants in a way similar to the acellular dermal matrix.

**Results:**

Overall complication rate was 14.9%. Capsular contracture occurred in 6.4%.There were no donor site complications. The majority of patients were satisfied with their cosmetic results. Sixty-eight percent rated their result as “excellent” or “good.”

**Conclusion:**

Thoracodorsal artery perforator flap can play a significant role in total breast reconstruction. In settings with limited resources, this flap can serve as an available autologous alternative to acellular dermal matrix.

## Background

Implant-based reconstruction is one of the most frequently used techniques for breast reconstruction after mastectomy. In 2008, an estimated 70% of all breast reconstructions performed in the USA were reliant on implants or tissue expanders [1].

This form of reconstruction is popular for its technical feasibility, short recovery, and good esthetic results. In addition, it is not associated with donor site morbidity. The implants are preferably placed in a subpectoral pocket, in order to decrease complications. The pectoralis major muscle is elevated from its inferomedial attachments, in order to optimize the shape of the pocket. A biologic material such as the acellular dermal matrix (ADM) is applied to extend the pocket inferiorly and laterally. ADM serves to achieve additional support and better definition of the lateral border and the inframammary fold [2].

The biologic materials such as ADM are not universally available. In many developing countries, there is a lack of financial resources hindering the provision of such material.

Thoracodorsal artery perforator (TDAP) flap has gained wide acceptance in recent years among reconstructive surgeons [3–5]. It is becoming popular for its versatility, reliability, and considerably low morbidity. Pedicled thoracodorsal artery perforator flap can be used as a pre-expanded manner (style), when needed in large soft tissue reconstruction [6, 7]. There are several reports describing its use in partial breast reconstruction. However, there are not as many reports of its use in total breast reconstruction [8].

In this study, we try to propose the TDAP flap as an autologous available alternative to ADM.

## Methods

This study was carried out after approval of the ethical committee of the National Cancer Institute of Cairo University.

The study is a prospective cohort of 47 patients, who were candidates for nipple-sparing mastectomy and were seeking immediate implant-based reconstruction. The research was carried out at the breast surgery unit of the National Cancer Institute of Cairo University between 2013 and 2015. The institute is a highly specialized academic institution. A constant team of two experienced breast surgeons and a highly experienced microvascular surgeon performed operative procedures.

Inclusion criteria for the study group were:

1. Breast cancer patients, who were candidates for nipple-sparing mastectomy: this included stage I or II breast cancer patients with tumor diameter less than 5 cm and distance to the nipple areola complex not less than 2 cm.
2. Patients with documented BRCA 1/2 mutations presenting for risk-reduction surgery.
3. Patients seeking implant-based reconstruction and consenting on harvesting the TDAP flap as an additional procedure.

Patients underwent:

- History and physical examination.
- Metastatic work-up for breast cancer patients.
- Preoperative counseling session by the operating surgeon to explain the operative procedure and expected complications.
- Handheld Doppler mapping and marking of the thoracodorsal artery perforators on the night before surgery.
- Preoperative photographing with a digital camera in three views: anteroposterior, oblique and lateral with arms to the sides, and elevated.
- Operative time was recorded.
- Patients came for follow-up 1 week, then 2 weeks postoperatively in the out-patient clinic. During these visits, postoperative photographs were taken in three views and all complications that have developed were recorded and dealt with.
- One year after the operation, patients were invited again to be reviewed by the operating surgeon where they were photographed in three views. They were asked to complete a five-scale questionnaire rating their cosmetic result as excellent, good, fair, poor, or very poor.
- Final pictures (in anteroposterior view) were processed by the BCCT.core20© software in order to obtain objective evaluation of cosmetic outcome [9].

The cosmetic results obtained by the BCCT.core20© software are based on the assessment of front views of patients for three main criteria: asymmetry between breasts, scar visibility, and color match.

In order to evaluate each criterion, several parameters are automatically measured in a patient’s photograph. For example, in order to judge the degree of asymmetry, the program calculates the difference of nipple position in each breast (the relative breast retraction assessment: pBRA), the level of the nipple compared to its counterpart (the relative upward nipple retraction: pUNR), the difference in the distance from each nipple to the inframammary fold (the relative breast compliance evaluation: pBCE), and finally, the difference in the surface area between breasts (the relative breast area difference: pBAD). Evaluation of scar visibility and color match occurs in a similar fashion. The program gives an overall result after automatically calculating all parameters for each criterion [9, 10].

### Technique

**Fig. 1:**
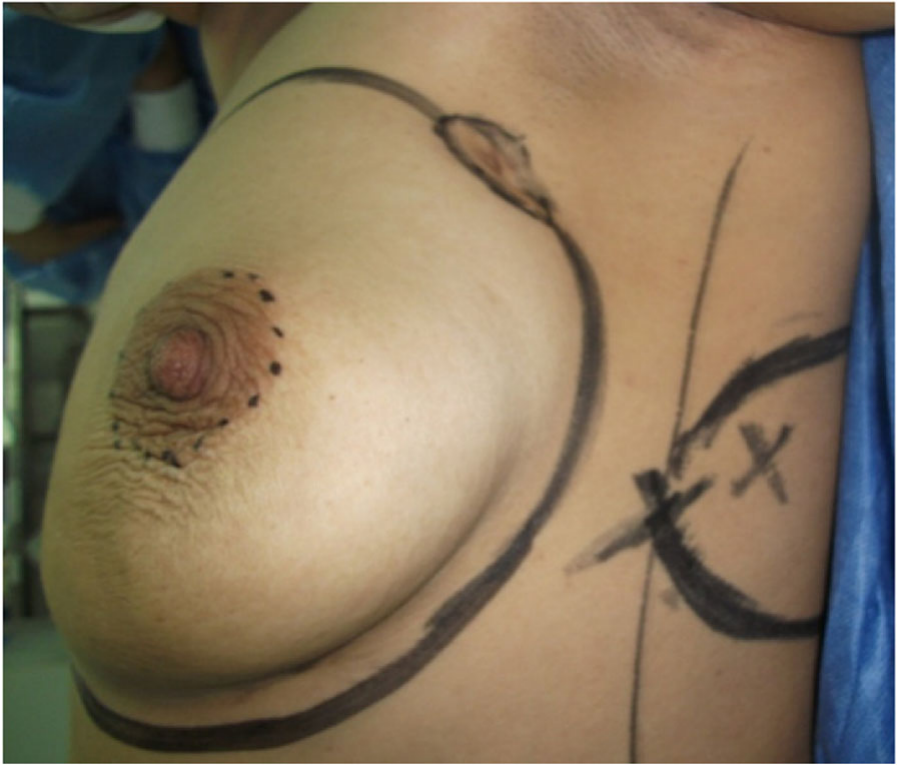
Preoperative markings with locations of perforator vessels marked as x

**Fig. 2:**
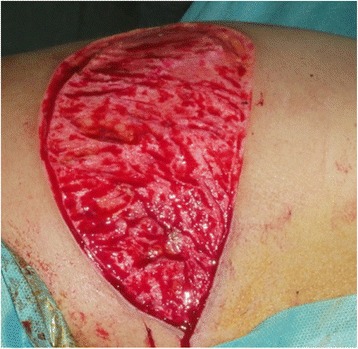
De-epithelialization of the skin paddle

**Fig. 3:**
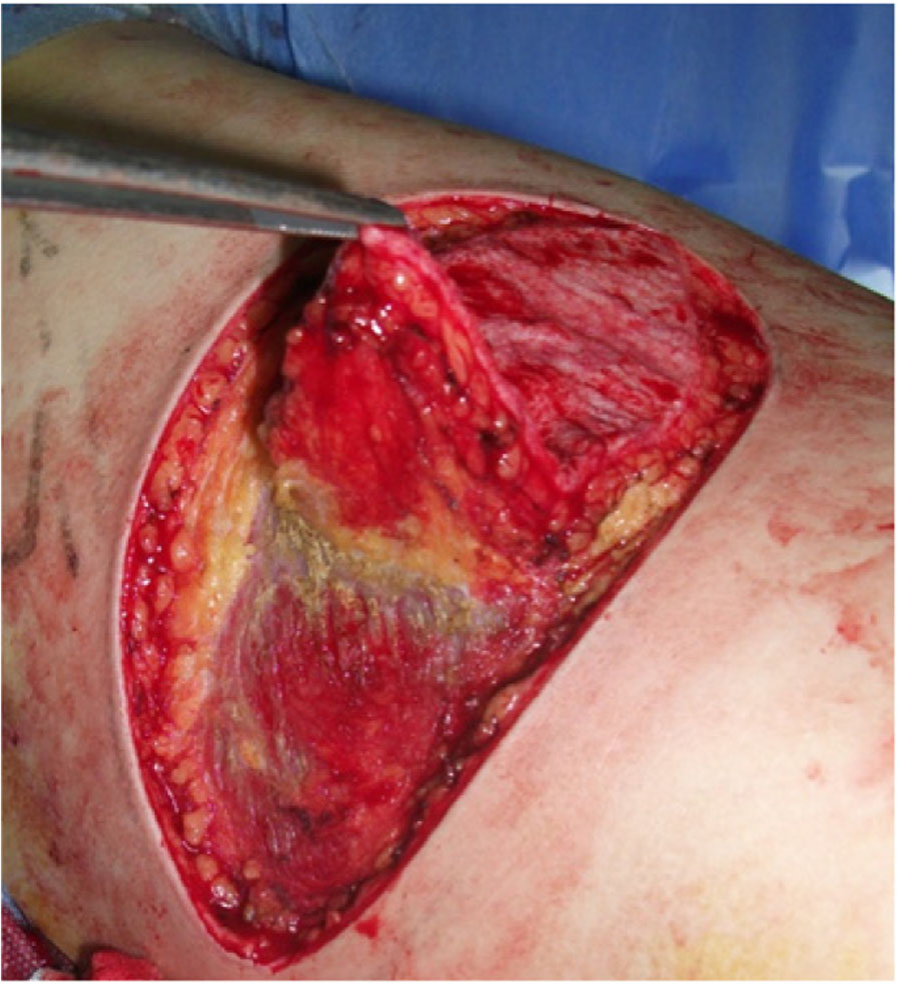
Flap elevation from distal to proximal above the fascia

**Fig. 4:**
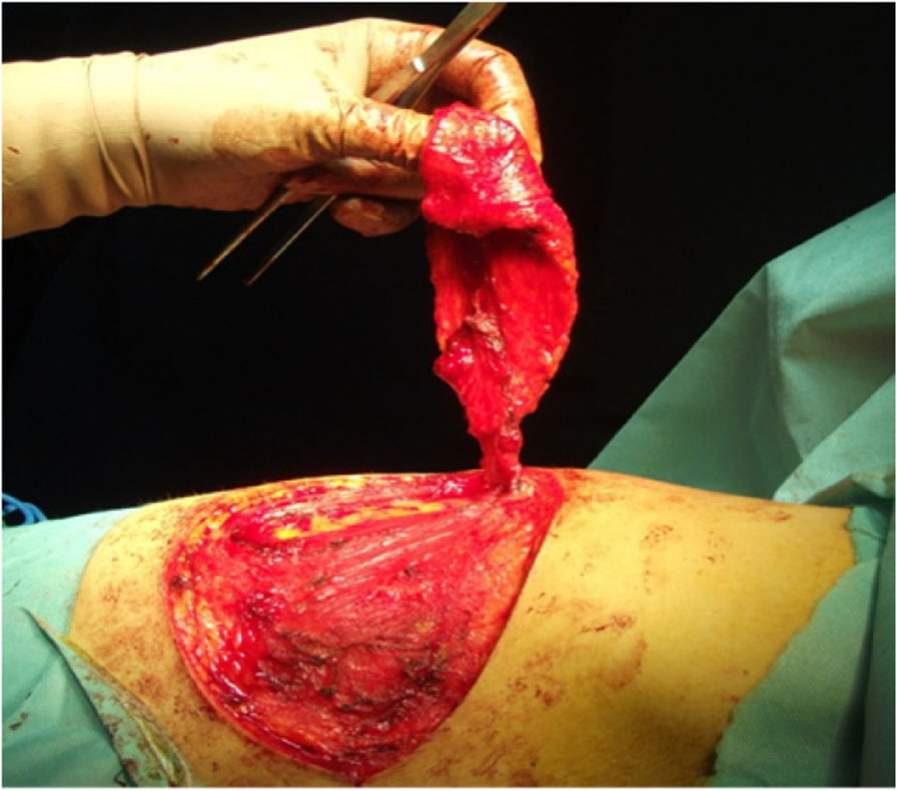
Flap harvest (usually with a piece of LD muscle around the perforator pedicle)

Based on the location of the Doppler-detected perforators, a skin paddle is fashioned in a way to contain the perforators in its most lateral part (Fig. 1). The skin over the flap is de-epithelialized (Fig. 2). Dissection then begins from distal to proximal in a plane above the fascia covering the latissimus muscle (Fig. 3). Bleeding points from the de-epithelialized skin paddle serve as good indicators of the diameter of the perforator vessels. Dissection continues until the perforator vessels are identified. A 2-cm piece of muscle is harvested around the perforator vessels in order to protect them (Fig. 4).

**Fig. 5:**
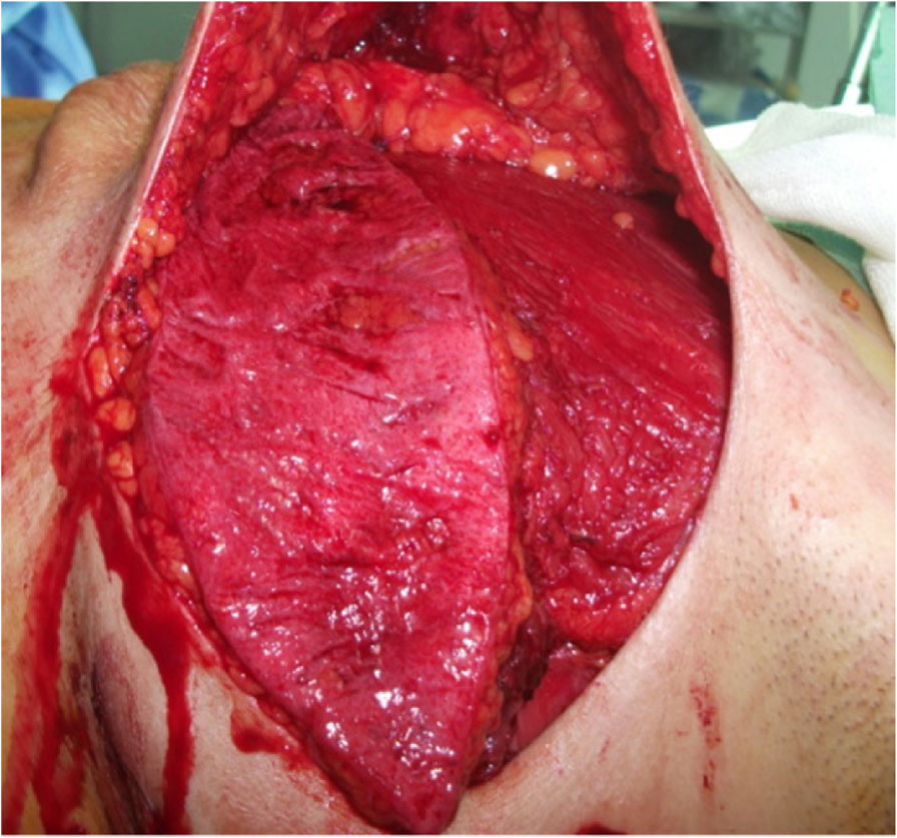
Flap rotated anteriorly 180°

**Fig. 6:**
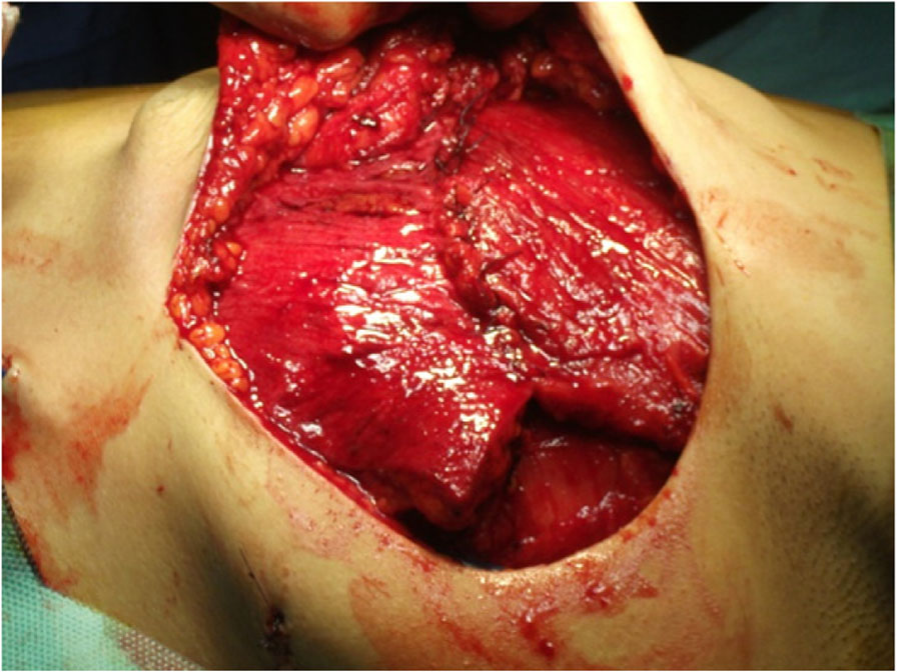
Pocket completed by suturing upper flap border to lower fibers of pectoralis major

The flap is rotated for 180° anteriorly (Fig. 5). In this manner, the upper border becomes inferior and the lower border assumes a superior position. After rotation, the upper border of the flap is sutured to the lower border and detached origin of the pectoralis major muscle (Fig. 6).

**Fig. 7:**
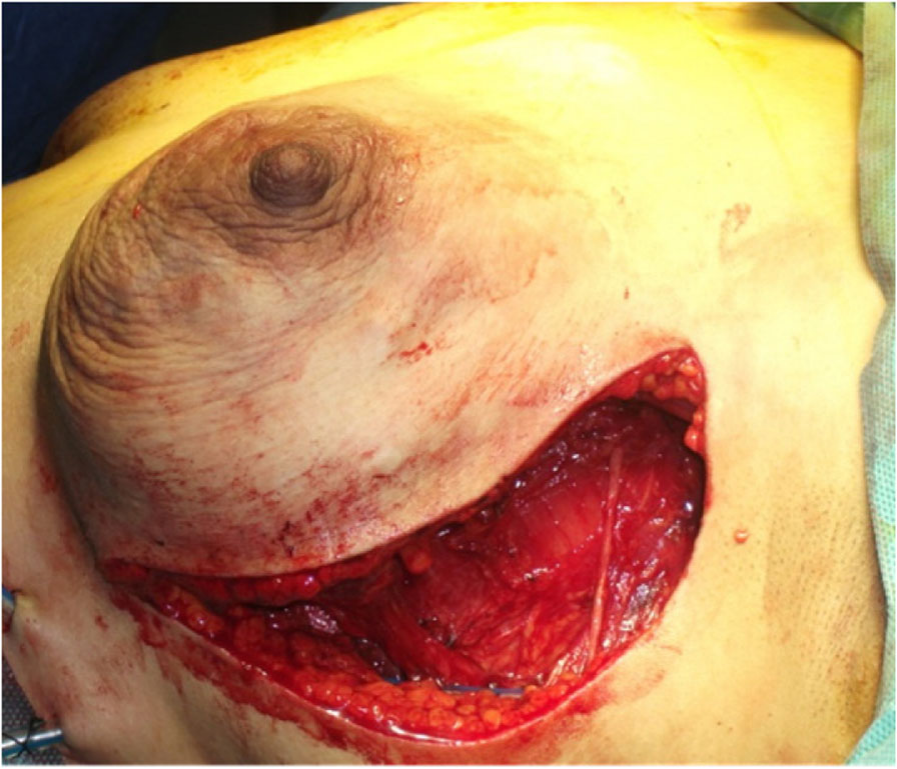
Implant placed in pocket

The lower border of the flap is sutured to the inner aspect of the inframammary fold. At this point, the pocket is complete and implant insertion follows (Fig. 7).

### Patients

The average patients’ age was 37 years (29–50). Two patients suffered from type II diabetes mellitus. Average BMI was 27.2 kg/m^2^ (range 23.4–31.1 kg/m^2^). There were 39 breast cancer patients and 8 women with documented BRCA 1/2 gene mutation, who presented for risk-reducing surgery. Thirty patients received postoperative radiation therapy. Average follow-up period was 30 months. (14–36 months).

## Results

The overall complication rate was 14.9%.There were two cases of superficial areolar sloughing (4.25%) and one case of superficial breast skin sloughing (2.13%). One patient suffered a postoperative hematoma that was evacuated (2.13%). The most common complication was grade II capsular contracture, which occurred in three patients (6.4%), all of whom received postoperative radiation. There were no donor site complications recorded.

Patients evaluated their cosmetic outcome as follows in Table 1:

**Table 1:** Cosmetic outcome (patients’ evaluation)

Excellent	Good	Fair	Poor	Very poor
7 (14.9%)	25 (53.1%)	14 (29.7%)	1(2.3%)	–

BCCT.core20© software showed the following cosmetic results displayed in Table 2:

**Table 2:** Cosmetic results according to BCCT.core20© software

Excellent	Good	Fair	Poor
4(8.5%)	27(57.5%)	16(34%)	

## Discussion

The main aim of this study was to demonstrate the technical feasibility and outcome of TDAP flap in total breast reconstruction. There are few reports with a limited number of patients that describe the use of this relatively new technique in total breast reconstruction [11, 12].

In this study, 47 patients underwent total breast reconstruction using TDAP flap and an implant.

A main challenge for the surgeon operating on implant-based reconstruction is to create a suitable pocket for the implant. A pocket will not only keep the implant in place but will also reduce the postoperative complications especially if the patient is to receive postoperative radiation.

At the same time, reconstruction of ptotic breasts is becoming more common as the age and body configuration of women seeking breast reconstruction continue to increase.

In patients where a subpectoral pocket is created, there has been an anatomical challenge as the pectoralis major muscle is only related to the upper two thirds of the breast. At the same time, the implant should be placed under muscle coverage as much as possible. Therefore, if the implant is placed in a strictly subpectoral pocket, it will result in a superiorly displaced breast mound, which does not respect the natural ptosis and the level of the inframammary fold. In addition, there will be an increased risk of upward implant migration.

In order to avoid this, surgeons have been using acellular dermal matrix (ADM) and other biosynthetic materials. These materials serve to expand the lower pole of the pocket and thus create a more “anatomically favorable” pocket with a better cosmetic outcome.

Another significant technique to protect the lower pole of the implant pocket is the dermal barrier flap [13]. This novel surgical technique has introduced the term skin-reducing subcutaneous mastectomy for the first time. It is designed as a skin-reducing/nipple-sparing mastectomy where the excess skin is not excised. Instead, the de-epithelialized lower pole of the breast skin is used as a dermal barrier flap to protect the inferior portion of the implant.

**Fig. 8:**
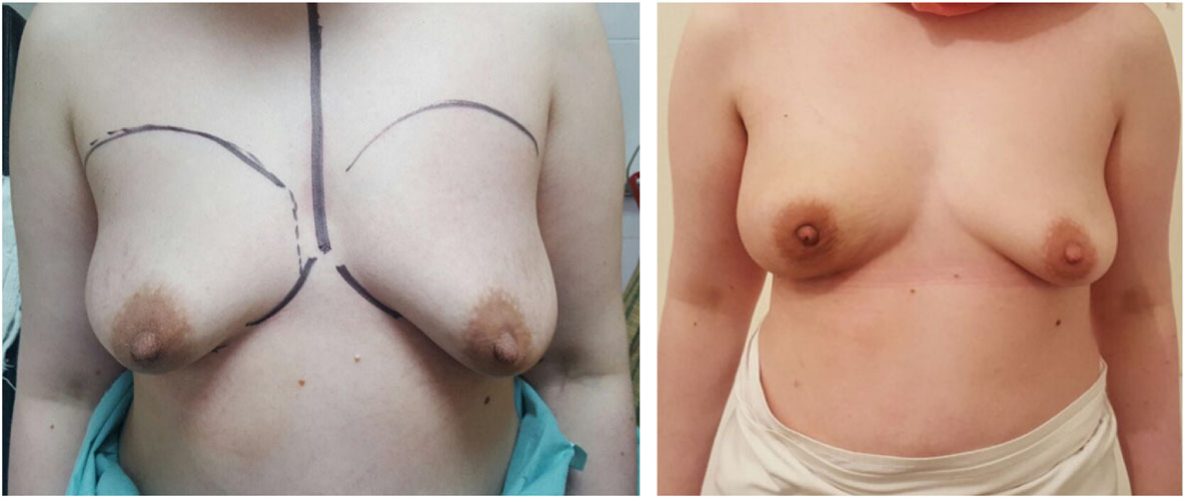
End result for a patient with right breast cancer after right nipple-sparing mastectomy

In developing countries, there are technological and financial restrains that make biotechnological materials, such as the ADM, unavailable to the reconstructive surgeons. In these settings, the TDAP flap can play an important role as an affordable autologous alternative to ADM. TDAP proved to be a reliable technique to complete the subpectoral pocket and support its inferior aspect. The flap enables the form-stable implant to rest in an “anatomically favorable” pocket that respects the natural ptosis and the level of the inframammary fold (Fig. 8).

**Fig. 9:**
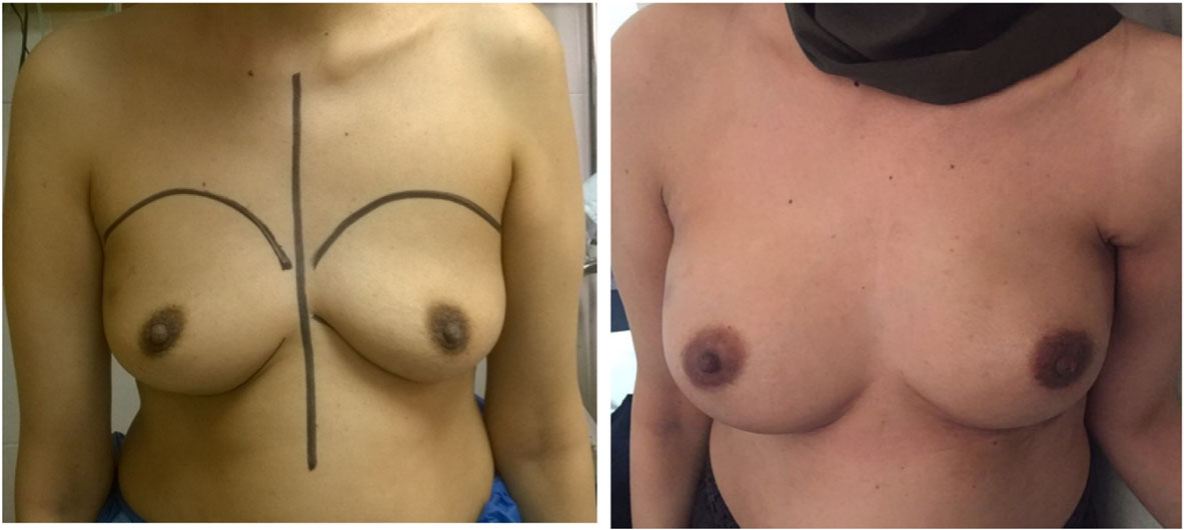
End result for a patient with BRCA 1 mutation after bilateral nipple sparing mastectomy

Maintaining the natural degree of ptosis is especially important to achieve symmetry in patients who refuse any surgical manipulation to the other breast (Fig. 9).

This technique showed acceptable outcome as regards complication rates and cosmetic outcome. The implant pocket created using this flap has shown a considerable degree of resilience to postoperative radiation. Three patients out of 30 who received postoperative radiation presented with capsular contracture.

The procedure showed an acceptable degree of patient satisfaction. Sixty eight percent of the study group have rated their results as excellent or good. Objective evaluation using the BCCT.core20© software showed a satisfactory cosmetic outcome where 66% of patients had a favorable rating.

Further prospective randomized research is warranted to compare this autologous technique to the ADM as regards cost effectiveness and overall outcome.

## Conclusion

Thoracodorsal artery perforator flap can be safely used in implant-based breast reconstruction. The technique achieved an acceptable outcome as regards complication rate and patient’s satisfaction. In low budget settings, this flap can be used as an autologous alternative to acellular dermal matrix.
